# Mosaic Genome Architecture of the *Anopheles gambiae* Species Complex

**DOI:** 10.1371/journal.pone.0001249

**Published:** 2007-11-28

**Authors:** Rui Wang-Sattler, Stephanie Blandin, Ye Ning, Claudia Blass, Guimogo Dolo, Yeya T. Touré, Alessandra della Torre, Gregory C. Lanzaro, Lars M. Steinmetz, Fotis C. Kafatos, Liangbiao Zheng

**Affiliations:** 1 European Molecular Biology Laboratory, Heidelberg, Germany; 2 Faculté de Médicine, de Pharmacie et d'Odonto-Stomatologie, Université du Mali, Bamako, Mali; 3 Dipartimento di Scienze di Sanità Pubblica, Sezione di Parassitologia, Università degli Studi di Roma-La Sapienza, Roma, Italy; 4 Department of Pathology, Microbiology and Immunology, School of Veterinary Medicine, University of California at Davis, Davis, California, United States of America; 5 Section of Infection and Immunity, Faculty of Natural Sciences, Imperial College London, London, United Kingdom; 6 Shanghai Institute of Plant Physiology and Ecology, Shanghai, China; Indiana University, United States of America

## Abstract

**Background:**

Attempts over the last three decades to reconstruct the phylogenetic history of the *Anopheles gambiae* species complex have been important for developing better strategies to control malaria transmission.

**Methodology:**

We used fingerprint genotyping data from 414 field-collected female mosquitoes at 42 microsatellite loci to infer the evolutionary relationships of four species in the *A. gambiae* complex, the two major malaria vectors *A. gambiae sensu stricto* (*A. gambiae s.s.*) and *A. arabiensis*, as well as two minor vectors, *A. merus* and *A. melas*.

**Principal Findings:**

We identify six taxonomic units, including a clear separation of West and East Africa *A. gambiae s.s.* S molecular forms. We show that the phylogenetic relationships vary widely between different genomic regions, thus demonstrating the mosaic nature of the genome of these species. The two major malaria vectors are closely related and closer to *A. merus* than to *A. melas* at the genome-wide level, which is also true if only autosomes are considered. However, within the *Xag* inversion region of the *X* chromosome, the M and two S molecular forms are most similar to *A. merus*. Near the *X* centromere, outside the *Xag* region, the two S forms are highly dissimilar to the other taxa. Furthermore, our data suggest that the centromeric region of chromosome 3 is a strong discriminator between the major and minor malaria vectors.

**Conclusions:**

Although further studies are needed to elucidate the basis of the phylogenetic variation among the different regions of the genome, the preponderance of sympatric admixtures among taxa strongly favor introgression of different genomic regions between species, rather than lineage sorting of ancestral polymorphism, as a possible mechanism.

## Introduction

A clear delineation of the divergence of closely related species is critical for understanding the process of speciation. However, reconstruction of historical relationships between closely related species is not a simple task, because these species usually have low levels of genetic divergence, and it is difficult to ascertain whether the recent divergence is the consequence of lineage sorting of ancestral polymorphisms or introgressive hybridization. In addition, the ‘gene tree versus species tree’ problem adds to the difficulty in establishing evolutionary relationships [1].

The *Anopheles gambiae* species complex consists of at least seven morphologically indistinguishable and largely sympatric (geographically co-existing) species [2], [3], [4], [5], [6]. *A. gambiae sensu stricto* (*s.s.*) and *A. arabiensis* are major malaria vectors, widely distributed in sub-Saharan Africa and are freshwater species. *A. quadriannulatus* species A and B are also freshwater breeders, but are not vectors, because of their strong zoophily and exophily. *A. merus* and *A. melas* are saltwater breeders and are mostly zoophilic but also bite humans in the absence of animals. *A. bwambae* breeds only in mineral water springs in Uganda and has a role as a local vector in this area. Laboratory crosses produce fertile female and sterile male offspring among all species pairs, and interspecies hybrids are found in nature at a low rate, of about 0.01–0.2% [2], [7], [8].

The pioneering work of Coluzzi *et al*. [3], [6] involved using the distribution of fixed inversions observed in polytene chromosomes to infer phylogenetic relationships within the *A. gambiae* species complex, on the assumption that cytologically identical inversions are monophyletic. *A. gambiae s.s.* and *A. merus* are both fixed for a cytogenetically identical inversion, *Xag*, and thus appear to be sister taxa, whereas *A. arabiensis* seems more distantly related as it lacks *Xag* and possesses three different inversions (*bcd*) that extensively overlap the region of the *Xag* inversion. The close phylogenetic relationship between *A. gambiae s.s.* and *A. merus* was further supported by sequence information from the *guanylate cyclase* gene on chromosome-*X* [9] and from the breakpoint region of the *2La* inversion [10]. However, contradictory results were obtained with other molecular markers. Indeed, rDNA and mtDNA sequence analyses suggested that *A. gambiae s.s.* is more closely related to *A. arabiensis* than to *A. merus* and *A. melas* [11]. Selective introgression has been observed in laboratory crossing of *A. gambiae s.s.* and *A. arabiensis*, in which the fate of chromosomal inversions was followed [12], [13]. Heterospecific *X*-chromosomes are lost very quickly, whereas some autosomal inversions can be introgressed quite freely under laboratory conditions [12]. To reconcile these conflicting datasets, Caccone *et al*. [14] proposed that the *A. gambiae* genome has a mosaic nature resulting from introgression between species in some chromosomal regions but not in the others. A similar architecture has been observed in species such as *Drosophila*, and can generate conflicting phylogenetic trees for markers at different regions of the genome [1]. Besansky *et al*. have provided evidence for different phylogenetic relationships in different genomic regions of *A. gambiae s.s.*, and implied that this species may have acquired traits from other sibling species, facilitating its spread and role as a main vector for human malaria in Africa [15].

In addition to the existence of seven sibling species of the *A. gambiae* complex, an incipient speciation process has been hypothesized within *A. gambiae s.s*. Based on non-random associations of several polymorphic chromosomal inversions, five chromosomal forms named Mopti, Savanna, Bamako, Forest and Bissau have been identified in *A. gambiae s.s.* [8], [16]. Field investigations in Mali have documented reproductive isolation of the Mopti, Savanna and Bamako chromosomal forms [8], [17]. However, laboratory crosses between these forms produce fertile offspring [8]. Analysis of molecular markers has revealed two molecular forms: M and S [18], [19], [20]. Comparison of molecular with chromosomal forms indicates that in Mali and Burkina Faso, the M-form corresponds to the Mopti chromosomal form, whereas the S-form encompasses the Savanna and Bamako chromosomal forms [18]. However, this correlation between chromosomally-defined and molecularly-defined forms does not apply in more humid southern areas [18], [19].

Two chromosomal regions have been found to significantly distinguish the M and S forms, i.e. near the *X*- and *2L*-chromosome centromeres. By using a set of 25 microsatellite loci distributed throughout the genome, it was shown that the M and S forms are practically indistinguishable except at two loci, *AGXH678* and *AGXE614*, both of which are outside the *Xag* inversion and close to the *X*-chromosome centromere and the rDNA locus that originally defined these forms [21]. This has been further supported by studies of 17 *X*-chromosome localized microsatellites [22], sequencing of 22 introns [23], and hybridization to Affymetrix gene expression arrays [24]. At locus *AGXH678*, different genotypes have also been observed between the M and S forms in another genome-wide study of 11 microsatellites [25]. In addition to the differences near the *X* centromere, allelic variation in the gene encoding a sodium-gated channel gene (*kdr*), close to the *2L* centromere that confers resistance to pyrethroids, is not equally distributed in the M- and S-forms [18], [26], [27], [28]. Variations in the *kdr* gene and in another region on chromosome *2R* were also detected in a genome-wide array comparison between the M- and S-forms [24]. Recently, sequencing results further confirmed differentiation of the M and S forms in regions close to the *X*- and *2L*-centromeres, and variation was reported on the *2R* chromosome between mosquitoes collected in Cameroon and Mali [29]. Subdivision within the M-form has been reported based on 12 microsatellites on the third chromosome [30].

To date, the analysis of the structure of the *A. gambiae* complex has been limited largely to single genes [9], [10], [14], [27], [28], or either to the *X*- [22], [23] or to the third- chromosome [30], and only preliminary genome-wide studies between the M- and S-forms have been carried out on 171 mosquitoes by pairwise comparison at 25 markers [21], and by using Affymetrix array hybridization of 14 individuals [24]. A genome-wide analysis of the *A. gambiae* complex in a large sample set is still lacking.

In this study, 414 field collected mosquitoes, representing both sympatric and allopatric samples, were genotyped at 42 microsatellites loci distributed throughout the genome, and the data were evaluated using three statistical methods. The results provide strong evidence in support of a mosaic genome architecture in the *A. gambiae* complex. A new taxonomic unit was identified within the S molecular form. Notably, a centromeric region of chromosome *3* stands out from our data as a strong discriminator between major and minor malaria vectors.

## Results

### (a) Variation among 42 microsatellites loci

**Figure 1:**
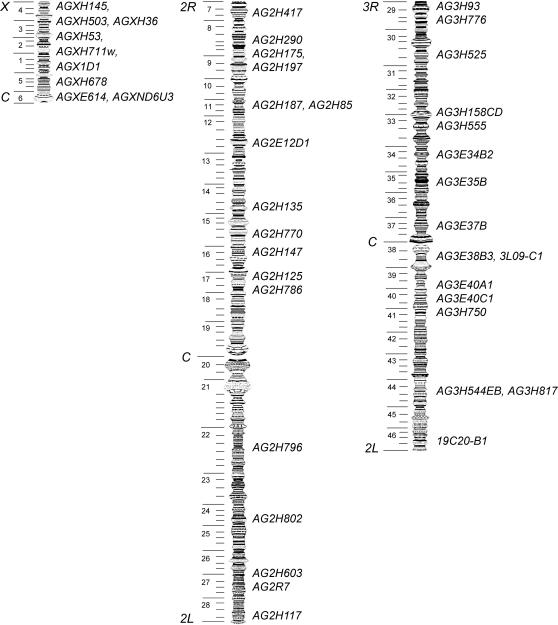
The 42 microsatellite markers used in the current study.

To characterize variation at multiple loci, the 414 individuals were grouped into nine populations (M1, M2, S1, S2, S3, A1, A2, R and L), according to species and collection sites (Table 1), and were genotyped at 42 microsatellite loci (Figure 1 and Table S1. For raw genotyping data, see Table S2). All 42 markers were either cytogenetically localized or assigned to chromosomal positions by comparison with the genome sequence [31]. PCRs were performed for each individual at each locus. All of the *A. gambiae s.s.* microsatellite primers gave PCR products in all nine populations (Table S3), except at six loci: two close to the *X* telomere (*AGXH145* amplifiable only in *A. gambiae s.s.* and *AGXH36* not amplifiable in either *A. arabiensis* or *A. melas*) and four near the centromere of chromosome *3* (*AG3E35B*, *AG3E37B*, *AG3E38B3* and *3L09-C1*). Interestingly, loci *AG3E35B* and *AG3E37B* could not be amplified in *A. merus* and *A. melas*, indicating significant sequence divergence from the major malaria vectors in these two minor vector species. In most cases the microsatellite loci were polymorphic among all species, except for one monomorphic locus in *A. merus* (*AG3H93*) and five loci in *A. melas* (*AG2H197*, *AG3H555, AG3E34B2, 3L09-C1* and *AG3E40A1*). Remarkably, four of these markers that are monomorphic in all 37 *A. melas* individuals, are located on the third chromosome, although insufficient sampling could not be ruled out as a cause of this observation.

**Table 1:** Origin of the 414 mosquito samples.

Species	ID	No.	Year	Burkina Faso	Mali			Kenya	Senegal
				Goundry	Sel	Soul	Kn	Kilifi	–
*A. gambiae s.s.* M molecular form	M1	24	1998	24					
	M2	80	1996		63	5	12		
*A. gambiae s.s.* S molecular form	S1	69	1996			69			
	S2	66	1996				66		
	S3	23	1998					23	
*A. arabiensis*	A1	20	1996		1	4	15		
	A2	61	1998					61	
*A. merus*	R	34	1998					34	
*A. melas*	L	37	1997						37

Species and forms of the *A. gambiae* complex are listed in the first column, and they are grouped into nine populations (ID), with the number of individual's (No.) and the year of collection (Year). The three villages in Mali are Selenkenyi (Sel), Soulouba (Soul) and Kokouna (Kn), respectively. “**–**” stands for missing information.

To identify potential differences among the nine populations, polymorphisms were characterized in terms of allele size distribution, allele size range, number of alleles and degree of heterozygosity at each locus (Figure S1 to Figure S5, and Table S3). In this way, shared or non-shared genetic variation among the nine populations could be explored at each locus. This analysis allows the identification of populations with similar patterns of genetic variation, which are thus expected to be more closely related genetically. For example, at locus *AGXH53*, the allele size ranges of *A. arabiensis*, *A. merus* and *A. melas* do not overlap, whereas those of the *A. gambiae* M- and S-forms do overlap with those of *A. arabiensis* and *A. merus*. Furthermore, at this locus the allele size distribution is relatively wide in M and S populations, as well as in *A. arabiensis*, but tightly clustered in *A. merus* and *A. melas*. The existence of non-overlapping alleles in certain populations suggests that these populations are distantly related compared with those possessing shared alleles in the regions covered by these markers.

As reported previously [21], the Mali M and S molecular forms (M2, S1 and S2) are quite comparable in terms of allele size distribution, differing significantly only at two loci, *AGXH678* and *AGXE614*. In this study, this was further confirmed at a third locus *AGXND6U3* (Figure S1), and also for the Burkina Faso M-form (M1), as well as for the Kenya S-form (S3) at these three loci. At locus *AGXH678*, for example, heterozygosities are lower in M populations (M1 = 29%and M2 = 34%) than in S populations (S1 = 86%, S2 = 92% and S3 = 65%). Heterozygosity does not correlate with sample size, indicating greater genetic variability at this locus in the S-form (Table S3).

### (b) Population Structure

**Figure 2:**
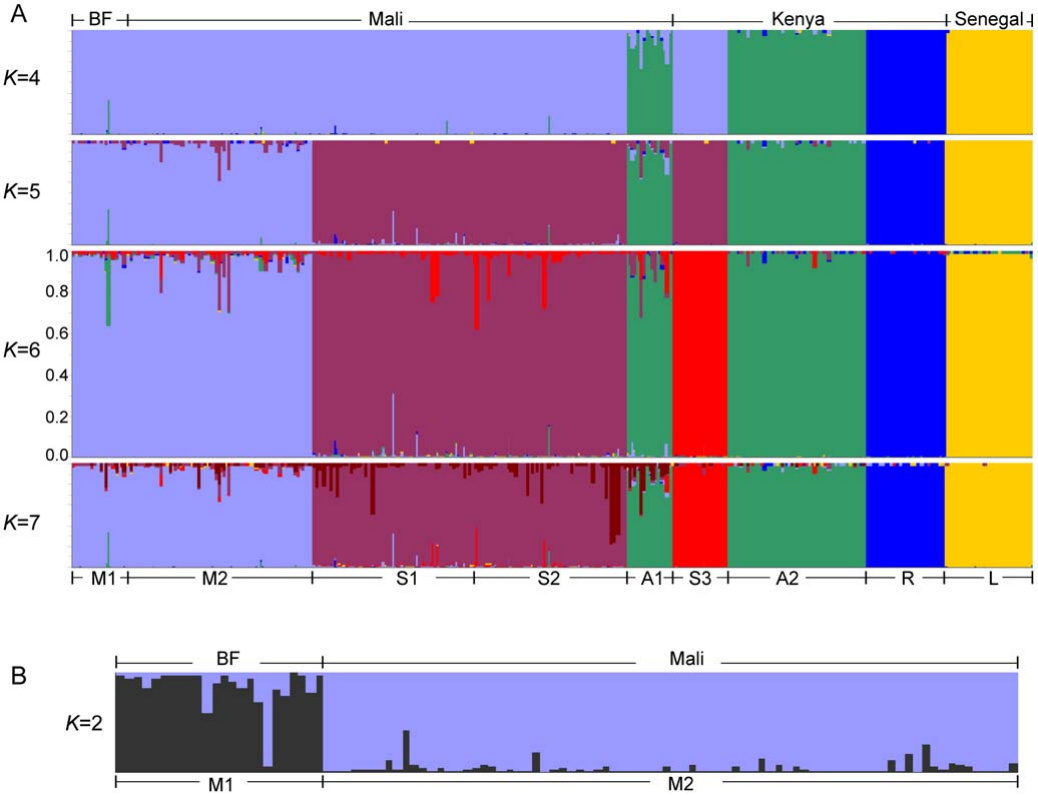
The *STRUCTURE* results. Consistent *STRUCTURE* results for 4≤*K*≤7 across the simulation lengths (5×10^5^, 10^6^ and 2×10^6^) for plot A. Each of the 414 individuals in the analysis is represented by a thin vertical line, and is partitioned into *K* colored segments that represent the individual's probability of belonging to one of the *K* populations. Mosquitoes were positioned in the figure according to the collection site (Burkina Faso (BF), Mali, Kenya and Senegal) and to their species/forms (M molecular form (M1 and M2), S-form (S1, S2 and S3), *A. arabiensis* (A1 and A2), *A. merus* (R) and *A. melas* (L). In plot B, the M1 is further separated from M2 for *K* = 2 with simulation length 5×10^5^.

Clustering analysis was performed on 36 loci spanning the genome. Of the initial 42 markers, six were excluded since they could not be amplified in all populations (Table S3). We used the program *STRUCTURE* [32], in which each individual is assigned a probability (i.e. the mean estimated ancestry) of belonging to one of *K* populations, where *K* is predefined in the simulation. Various combinations of the number of populations *K* (1≤*K*≤10) and the simulation lengths (10^4^, 5×10^4^, 10^5^, 5×10^5^, 10^6^ and 2×10^6^ “Burn-ins” and “Repeats” each) were tested with three different input orders for the individuals. Consistent results were obtained for 4≤*K*≤7 with long simulation length (5×10^5^, 10^6^ and 2×10^6^), independent of input order (Figure 2A and for *K*≤3 or *K*≥8, data not shown), suggesting that short simulation lengths (10^4^, 5×10^4^ and 10^5^) are inadequate for our application (data not shown). When *K* is 4, four clusters, corresponding to the four species, i.e. *A. gambiae s.s.*, *A. arabiensis*, *A. merus* and *A. melas*, could be distinguished. When *K* is 5, the M and S molecular forms were further separated (Figure 2A). Interestingly, when *K* is 6 or 7, six distinct clusters were obtained. S mosquitoes were further separated into two clusters: S1 and S2, both from Mali, and S3 from Kenya, implying possible new taxonomic units within the S molecular form that correlate with spatial isolation. When we ran *STRUCTURE* with *K* set to 7 using 25 of the 42 microsatellites, i.e. those we had used in our previous study [21], clustering of the six units was not observed (data not shown), suggesting that these 25 markers are not adequate for a fine-scale phylogenetic study of the *A. gambiae* complex, particularly when one aims to characterize the closely related *A. gambiae s.s.* M and S molecular forms.

The logarithmic value of probability distribution *ln(P(X/K))* and the probability of a given range of clusters *P(K/X)* were listed in Table 2, for cluster number calculation. The value of *ln(P(X/K))* was the output of the *STRUCTURE* analysis, where *X* denotes genotypes of the sampled individuals for the K cluster. *P(K/X)* was calculated on the basis of *ln(P(X/K))* value by considering *K* = 3,…,8 [32]. The largest value of *P(K/X)* corresponds to the smallest *ln(P(X/K))* in the given range of *K*. Consistent results were obtained across independent runs (5×10^5^, 10^6^ and 2×10^6^), as well as all three different initial input orders (for the other two sets of calculations with different input orders, data not shown). For *K* = 6, the probability was 1 (Table 2). Therefore, we conclude that the most likely number of extant taxonomic units is six in the sample set, i.e., the M molecular form, the Mali and Kenya S forms, *A. arabiensis*, *A. merus* and *A. melas* (Figure 2A).

**Table 2:** Inferring the number of clusters.

*K*	*ln(P(X/K))*			*P(K/X)*		
	5×10^5^	10^6^	2×10^6^	5×10^5^	10^6^	2×10^6^
3	−57115	−57113	−57113	∼0	∼0	∼0
4	−55078	−55075	−55076	∼0	∼0	∼0
5	−53670	−53674	−54942	∼0	∼0	∼0
**6**	**−52907**	**−52905**	**−52907**	**1**	**1**	**1**
7	−53091	−53483	−55253	∼0	∼0	∼0
8	−54128	−55138	−52944	∼0	∼0	∼0

*A. gambiae* species complex (414 mosquitoes) at 36 microsatellite loci. For each *K* number of clusters of the *X* genotypes, the estimated values of *ln(P(X/K))* of three independent runs (5×10^5^, 10^6^ and 2×10^6^ “Burn-ins” and “Repeats” each) are listed. For each simulation length, the probability number of clusters *P(K/X)* values were calculated by considering *K* = 3,…,8.

Finally, separate simulation runs using *STRUCTURE* were performed using only individuals with different geographical origins that we suspected might be subdivided. No subpopulations were identified in the Mali S-form (S1 and S2) and Mali and Kenya *A. arabiensis* (A1 and A2), but Burkina Faso (M1) and Mali M-form (M2) were further subdivided (Figure 2B and Table S4).

The advantage of *STRUCTURE* is that it can not only identify population structure, but also detect migrants or admixed individuals [32]. In our analysis, any mosquito attributed to one cluster but with a higher than 5% mean estimated ancestry probability of belonging to another cluster is considered to be a migrant or admixed individual, which represents a quantified indicator of gene flow (Table 3). Admixed individuals between M and S forms (14 sympatric and 5 allopatric individuals, respectively) and between *A. gambiae s.s.* and *A. arabiensis* (13 sympatric and 5 allopatric mosquitoes, respectively) are the most common ones. Two sympatric and one allopatric migrant were found for *A. merus* with the other taxa, and none for *A. melas*. The preponderance of sympatric admixtures (70% when all five taxa are considered) suggests that introgression is the main contributor to the observed similarities among taxa. However, nine allopatric migrants were observed between Mali and Kenya S forms, suggesting that the detected migrants between the two S forms may be the results of ancestral polymorphisms because of the high-level geographic separation.

**Table 3:** Migrants or admixed individuals detected between the taxonomic units.

Number (%) of migrants	M1	M2	S1	S2	S3	A1	A2	R	L
M molecular form	–	–	5 (3.0)	1 (0.6)	1 (0.8)	3 (2.4)	0	0	0
Mali S molecular form	0	8 (3.7)	–	–	1 (0.6)	7 (4.5)	1 (0.5)	0	0
Kenya S molecular form	0	4 (3.9)	2 (2.2)	6 (6.7)	–	2 (4.7)	1 (1.2)	0	0
*A. arabiensis*	2 (1.9)	1 (0.6)	0	1 (0.7)	0	–	–	0	0
*A. merus*	0	0	2 (2.0)	0	0	0	1 (1.1)	–	0
*A. melas*	0	0	0	0	0	0	0	0	–

The absolute number of migrants is indicated. The percentage of migrants relative to the total number is given in brackets.

### (c) Evidence for a mosaic genome in the *A. gambiae* complex

The recombination rate varies for different regions of the genome, especially for the *X* chromosome of *A. gambiae* [33], [34], making it inappropriate to compare averages genetic distance measurements (e.g. *Fst*) across the genome when analyzing multilocus genotyping data. Therefore, we used systematical principal component analysis (PCA) and correspondence analysis (CA), where portions of the genome each comprising a few markers were analyzed to further clarify the phylogenetic relationships of the *A. gambiae* complex. At each locus, mean allele sizes for each genotyped individual were used.

PCA reduces the dataset dimension and enables visualization of individuals and populations in 3D or 4D graphics. The first principal component (PC) represents the largest part of the variance in the whole dataset (i.e. corresponding to the most separated cluster), followed by the second largest part, the third, etc. CA involves two separated PCA analyses that are merged to represent the rows (population) and the columns (microsatellite marker) of a two-dimensional matrix [35]. As a result, CA can also reveal correlations between populations and microsatellite markers that are plotted on the same graphs.

**Figure 3:**
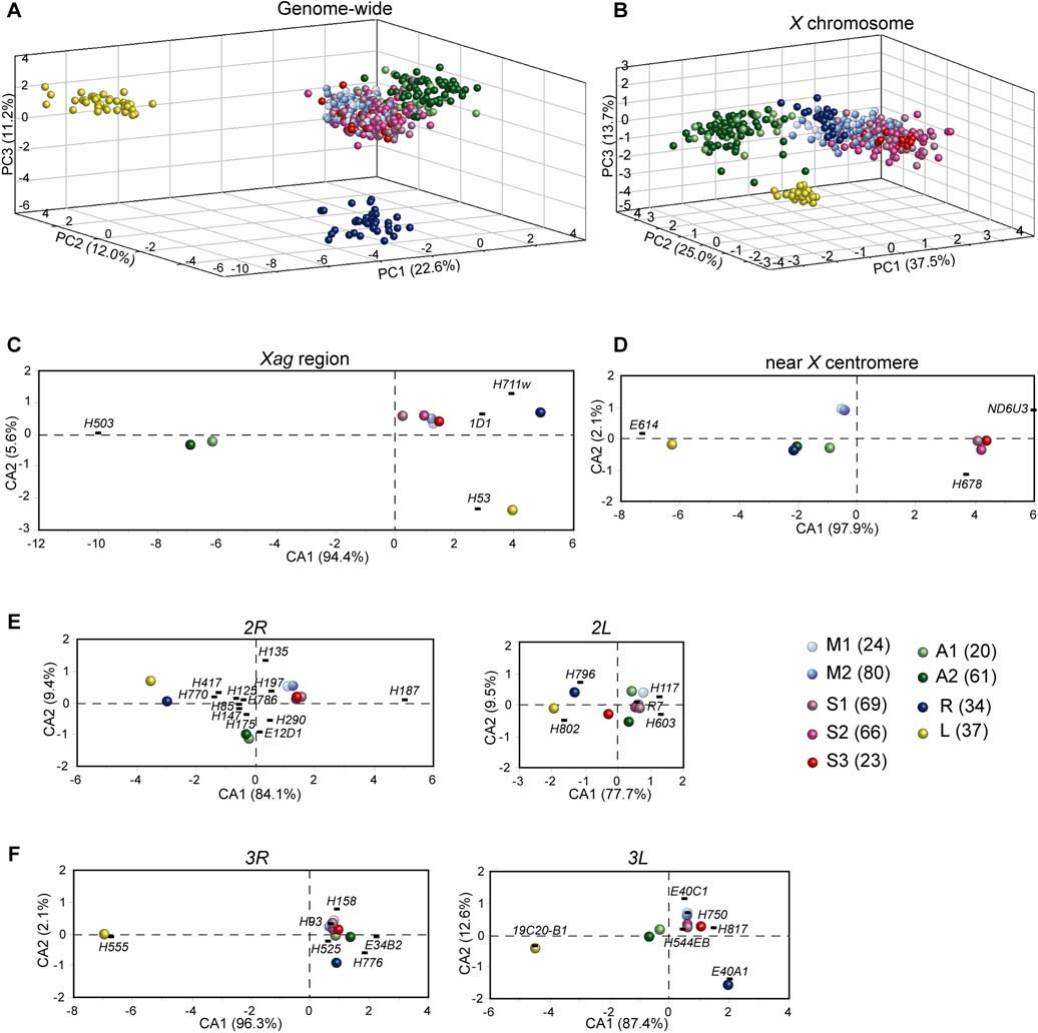
PCA and CA results of the 414 individuals at 36 loci. PCA analysis of the 414 individuals in a genome-wide study for 36 microsatellites (A) and for seven loci on the *X* chromosome (B). CA analysis of the nine populations in the *Xag* inversion region (C), near the *X* centromere region (D), on chromosomes *2* (E) and *3* (F). Individuals and populations are color-coded as described (see legend where numbers in brackets next to the populations name indicate the number of mosquitoes in each group). The contribution of each PC or CA is indicated as a percentage in brackets on the axis. In plots A and B, each point represents a single mosquito, while in plots C to F, each point represents a population. In plots C to F, microsatellite markers are localized by black bars. The first three letters of their name (AGN, where N is chromosome *X*, *2* or *3*) were removed.

Each PC is a weighted sum of microsatellites, the weights representing the strength of the microsatellite contributions to the PC. First, we analyzed the 414 individuals at 36 loci. The first principal component (PC1) separated the two major vector species (*A. gambiae s.s.* and *A. arabiensis*) from the zoophilic species (*A. merus* and *A. melas*) (Figure 3A and Figure S6A). PC1 was dominated by eight loci across the genome (two, four and two from chromosomes *X*, *2* and *3*, respectively; Table 4A), indicating that eight loci are mainly responsible for separating the two major and the two minor vectors in our sample set. PC2 (dominated by three, two and three loci from chromosomes *X*, *2* and *3*, respectively) and PC3 (dominated by three, two and three markers from chromosomes *X*, *2* and *3*, respectively) further distinguished the four named species, *A. gambiae s.s.*, *A. arabiensis*, *A. merus* and *A. melas*, from each other. Finally, PC4 separated the M and S molecular forms (Figure S6A); it was dominated by six loci: *AGXH678*, *AGXE614* (both near the *X* centromere) and four from chromosome *2*.

**Table 4:** The weights of the microsatellites contributing to the principal components.

Chromosome	Markers	A: *A. gambiae* complex				B: *A. gambiae s.s.*		
		PC1	PC2	PC3	PC4	PC1	PC2	PC3
*X*	AGXH145	-	-	-	-	0.8	**15**	0
	AGXH503	1.1	3.5	**11**	0.2	0.5	2.5	4.2
	AGXH36	-	-	-	-	0.1	**11**	**5.1**
	AGXH53	**5.6**	1.9	**6.7**	0.6	0.1	1.2	0.1
	AGXH711w	1.6	**6.4**	2.5	2.7	1.4	0.2	0.3
	AGX1D1	2.3	**6.3**	**6.3**	0.1	1.1	0.5	**6.5**
	AGXH678	1.2	1	0.2	**28**	**13**	0	0.5
	AGXND6U3	**6.3**	**5.9**	1.3	2.3	**12**	0.8	0
	AGXE614	3.6	0.2	1	**11**	**15**	0.1	0.3
*2R*	AG2H417	**6.5**	1.1	1.2	0.5	0.7	1.7	0
	AG2H290	**6.1**	0	4.3	0.4	3.3	0.1	1.2
	AG2H175	0.1	**8.1**	0.1	2.2	2.5	3.5	0.3
	AG2H197	1.9	4.3	1.5	2.6	1.8	1.6	1.2
	AG2H187	**6.2**	2.6	0	0.6	0.9	0.1	2.3
	AG2H85	0.8	0	4.2	**15**	**5.8**	0.3	0.5
	AG2E12D1	3.7	4.2	**6.8**	0.1	3.4	0.6	0
	AG2H135	0.1	**12**	3.5	2.3	**6.3**	0	0.3
	AG2H770	3.3	2.1	**9.7**	0.2	2	**12**	0.7
	AG2H147	2.4	0	1.4	**5.6**	0.6	0.8	3.3
	AG2H125	2.4	0	2.5	0.9	0.4	0	4.1
	AG2H786	1.1	2.2	0	0.3	0	**5.7**	4.2
*2L*	AG2H796	2.8	1	0.4	**7.9**	4	2.2	2.3
	AG2H802	1.3	0.9	0.2	**6**	1	2.9	1.8
	AG2R7	2.4	3.1	0.1	4	1.9	3.1	1.8
	AG2H603	2	0.1	2.4	0.3	0.2	1.2	0.4
	AG2H117	**9.4**	0.2	0.1	0.4	0.3	1.8	**16**
*3R*	AG3H93	0.1	**5.2**	3.2	0.1	0.1	0.8	**5**
	AG3H776	2.5	2.2	0.7	2	0.4	1.3	**5.9**
	AG3H525	0.2	1.4	0.4	0.3	0.1	4.5	**9.1**
	AG3H158CD	0.8	**7.6**	1.4	0.2	0.5	2.6	0.1
	AG3H555	**8.6**	3.6	0.8	0.1	0.2	0	0
	AG3E34B2	1.4	0.1	0.5	0.1	0	0.5	3.4
	AG3E35B	-	-	-	-	2.6	0	2
	AG3E37B	-	-	-	-	0.2	0.4	0.3
*3L*	AG3E38B3	-	-	-	-	4.1	3.1	0
	3L09-C1	-	-	-	-	4	1	3.1
	AG3E40A1	0	**7.9**	**12**	0.2	**7.7**	0.3	0
	AG3E40C1	0.6	3.3	0.8	0	0.1	1.9	0.1
	AG3H750	0.3	0.2	0	1	0.2	2.7	**6.1**
	AG3H544	2	0	0.1	0.4	0.1	**5.4**	1.2
	AG3H817	2.8	0.4	**7.5**	1.4	0.6	4.9	0
	19C20-B1	**6.5**	0.6	**5.1**	0.1	0	2.4	**5.9**
	Eigenvalues	8.1	4.3	4.1	1.7	4.4	1.9	1.7

The weights of the microsatellites contributing to the principal components in the PCA analysis of the 414 mosquitoes at 36 loci (A) and of 262 *A. gambiae sensu strictu* at 42 loci (B).The weights of the microsatellites were calculated by multiplying the corresponding eigenvectors and the bold values indicating bigger contribution of the locus to the principal components with higher than 5%.

When we focused our analysis only on the *X* chromosome, a different phylogeny was obtained. The M and S molecular forms of *A. gambiae s.s.* are separated from each other, and the M-form is closely related to *A. merus*, and is more distant from *A. arabiensis* and *A. melas* (Figure 3B and Figure S6B).

PCA analyses of all 414 mosquitoes taken either as individuals or as nine populations led to generally congruent results, differences were nonetheless found in the contributions to the total variance (Figure 3 and Figure S6). PCA transforms and reduces the 36 multidimensional dataset (for the 36 loci) to lower dimensions while retaining those characteristics of the dataset that contribute most to PCA variance. The PC1, PC2 and PC3 contributed 22.6%, 12% and 11.2% to the total variance in the PCA of the 414 mosquito taken individually, respectively (Figure 3A). However, PCA of the nine populations showed a more efficient reduction of dimensionality, with the corresponding contributions of 45.4%, 24.4% and 16.7%, respectively (Figure S6C). Although PCA of individuals is less efficient for the analysis of the phylogenetic relationship among populations, it reveals variations within each population.

When studying the *Xag* inversion region in the nine populations using CA, we noticed that the M and S forms of *A. gambiae s.s.* were very much alike and closely related to *A. merus*, but were more distant from *A. melas* and *A. arabiensis* (Figure 3C), which is almost identical with the eigenvectorial representation based on the frequency of chromosome polymorphic inversions (see Figure S2A in [5]). These data indicate that, in the *Xag* region, the maximum affinity is between *A. gambiae s.s.* and *A. merus*, consistent with the hypothesis of a monophyletic inversion. This conclusion was reinforced by the observation that the locus *AGXH36*, which is also located within the *Xag* inversion, is amplifiable in *A. gambiae s.s.* and *A. merus*, but not in *A. arabiensis* or *A. melas* (Figure S1 and Table S3). However, the three loci near the centromere on the *X* chromosome outside the *Xag* region showed a very different pattern, which clearly separated the S-form from both the M-form and the other species (Figure 3D).

Markers on chromosome 2 (*2R*, 12 loci; *2L*, 5 loci) showed that *A. gambiae s.s.* is more closely related to *A. arabiensis* than to *A. merus* and *A. melas* (Figure 3E). Markers on chromosome 3 (*3R*, 6 loci; *3L*, 6 loci) indicated a separation of *A. melas* from the other species (Figure 3F), which might have resulted partially from the four invariant alleles in *A. melas*.

**Figure 4:**
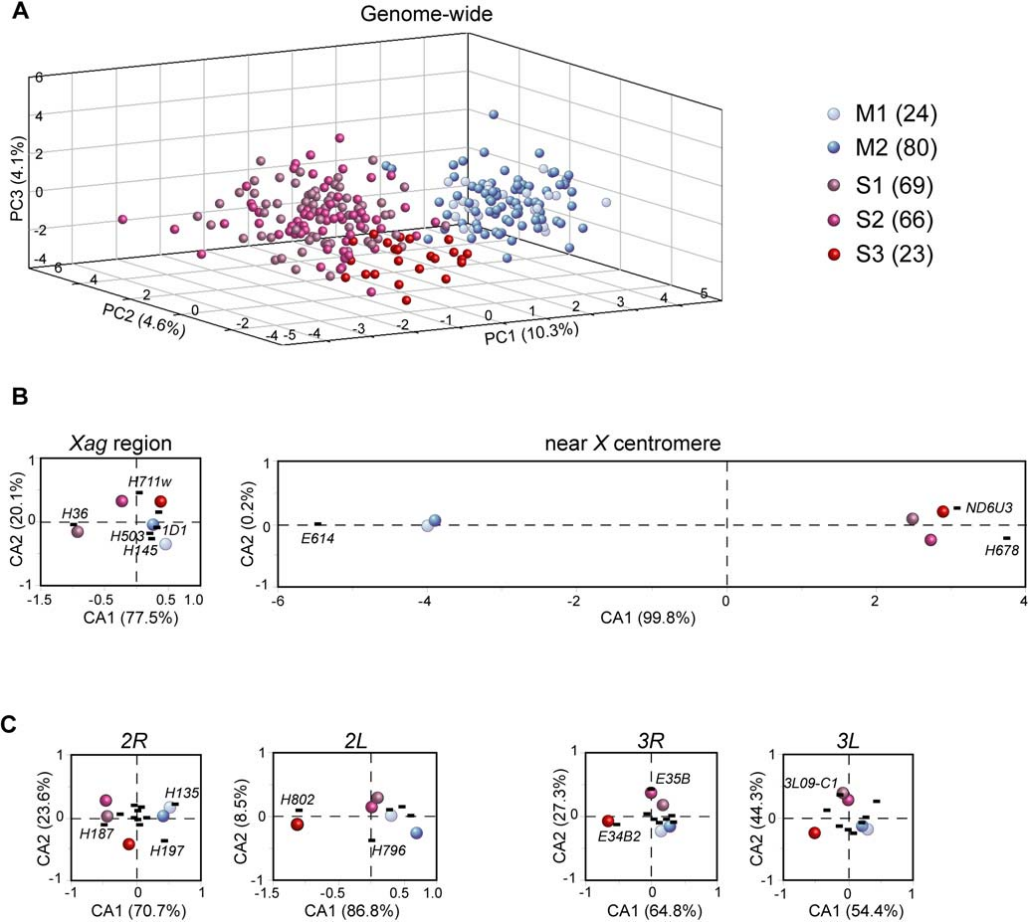
PCA and CA results of the 262 *A. gambiae s.s.* at 42 loci. PCA results of the 262 *A. gambiae s.s.* at 42 microsatellites (A), and CA results of the five populations of *A. gambiae s.s.* in the *Xag* inversion region and near the *X* centromere (B), and on autosomes (C). Each individual (in plot A) or each population (in plots B and C) is represented by a single point. See Figure 3 for legend. Only the most significant microsatellites markers are indicated.

Finally, PCA analysis of the 262 *A. gambiae s.s.* samples at 42 microsatellite markers, including the six loci *AGXH145*, *AGXH36*, *AG3E35B*, *AG3E37B*, *AG3E38B3* and *3L09-C1,* which could be amplified in all *A. gambiae s.s.* individuals but not in the other species, provided more detailed information about the phylogeny of *A. gambiae s.s.* (Figure 4A and Figure S6D). The M and S forms were separated by PC1 (dominated by *AGXH678*, *AGXND6U3*, *AGXE614*, *AG2H85*, *AG2H135* and *AG3E40A1*, see Table 4B). Mali and Kenya S forms were distinguished by PC2 and PC3, dominated by three, three and six loci on chromosomes *X*, *2* and *3,* respectively. To identify the chromosomal regions that are responsible for this separation, we applied CA to the mean genotypes of the five populations at each locus (Figure 4B and 4C). The Kenya S-form was shown to be markedly separated from the Mali S-forms across the whole genome, except in the region near the centromere of the *X* chromosome, outside the *Xag* inversion.

The correlations between taxa and microsatellite markers can be extracted from the CA graphs shown in Figures 3C–F and Figures 4B–C, which are more specific than the markers found by PCA (see above). For instance, the M-form is identified by loci *AG2H135* and *AG2H197*, the S-form by loci *AGXH678*, *AGXND6U3* and *AG2H187*, while loci *AG2H802* and *AG3E34B2* are more specific for Kenya S-form (S3). *A. arabiensis* is identified by loci *AGXH503* and *AG2E12D*, and *A. merus* by loci *AG2H796* and *AG3E40A1*. For *A. melas*, there are five most contributing loci, *AGXH53*, *AGXE614*, *AG2H802*, *AG3H555* and *19C20-B1*.

## Discussion

To investigate the phylogenetic relationships among species in the *A. gambiae* complex, we have used microsatellite polymorphism in 414 field-collected female mosquitoes with 42 markers covering the whole genome, and systematically analyzed the data genome-wide or regionally using three statistical methods: the program *STRUCTURE*, Principal Component Analysis and Correspondence Analysis. These three statistical methods are applicable for studying various individuals or populations, genome-wide or in parts of the genome. The *STRUCUTRE* method is more suited to whole genome phylogeny analysis (with a large number of loci) under proper simulation conditions, and has the great advantage of enabling the identification of the most likely number of extant populations, and for the detection of migrants or admixed individuals among these populations [32]. In contrast to *STRUCTURE*, PCA and CA are more determinate, and are not only capable of differentiating individuals or populations from each other, but also of identifying the genomic regions responsible for the observed differentiation, which makes them useful tools to detect and analyze mosaic genomes

Our results demonstrate an evident genome mosaicism within the *A. gambiae* complex. The two major malaria vectors (*A. gambiae s.s.* and *A. arabiensis*) are closely related, further away from *A. merus* and most distantly related to *A. melas*, in agreement with the analyses on rDNA and mtDNA [11]. This indicates that *A. arabiensis,* rather than *A. merus,* is the sister taxon of *A. gambiae s.s.*, in contrast to expectations based on chromosomal inversion patterns [5]. Nevertheless, within the *Xag* inversion on the *X* chromosome, the M and S molecular forms of *A. gambiae s.s.* are indeed most similar to *A. merus*, and more distant from *A. melas* and *A. arabiensis*. These results support the inference from polytene chromosomal inversions [3], [5] and agree with the single gene (guanylate cyclase) and *2La* marker studies [9], [10]. Outside the *Xag* region and near the *X* centromere, the S form is the most distant to the others, in particular to the M form, in agreement with previous results [21], [22], [23], [24]. In addition, the M form is equally close in this region to *A. merus* and to *A. arabiensis*.

Within *A. gambiae s.s.*, three taxonomic units, the M and two S molecular forms, have been identified in our sample set. The two S-forms are allopatric, consistent with the separation of the southeastern vs. northwestern S forms observed previously [25]. Considering the high-level of premating isolation of these allopatric mosquitoes, we can assume that the observed genetic divergences between the Mali and Kenya S forms are the direct consequence of geographic isolation. Interestingly, we did not observe a similar separation in two clusters for the Mali and Kenya *A. arabiensis* populations (A1 and A2), although they were collected from the same sites as the two S-forms. Moreover, the weaker separation of the Burkina Faso and Mali M forms is consistent with the results obtained using different statistical methods and concentrating on the third chromosome [30]. Still, as the sample sizes from the Burkina Faso M1, as well as from the Kenya S3 populations are small, the results obtained here need to be verified on larger samples of wild mosquitoes and by laboratory crosses.

In regards to the ‘gene tree versus species tree’ issue, it is generally accepted that the use of multiple loci would in principle provide a more complete picture of the divergence history for a group of closely related species [1]. The *A. gambiae* genome size is about 278 megabases (Mb) [31], leading to a coverage of the 42 microsatellites at about 7 Mb per marker, which represents the highest resolution to date. Previous studies from us and others, with 25 or fewer microsatellites were not as robust to resolve the phylogeny of the *A. gambiae* complex at the genome-wide level, especially among the closely related molecular forms [21], [25]. This might be due to the lower number of markers used compared to our study, and/or to a “biased” choice of markers, although the 25 microsatellites used in [36] were evenly distributed throughout the genome. Moreover, in contrast to previous reports that relied on the analysis of each locus separately, we used PCA and CA methods that can grasp the different contributions of multiple loci, and therefore allow a true genome-wide comparison of the different populations.

Our analysis also identified genomic regions that are characteristic of certain populations or taxa. First, despite the fact that polymorphisms are shared among the nine populations overall, four loci near the centromeric region of chromosome *3* exhibit private alleles for all 37 *A. melas* individuals. This observation awaits further validation with samples from different collection sites and of larger sizes. Second, the two minor malaria vectors can be distinguished from the two major vectors at two loci in the centromeric region of chromosome *3*. This region is therefore a potential strong discriminator between major and minor malaria vectors. This differentiation might be related to ecological factors as well, such as fresh water versus salt water breeding habitats, which distinguish the major vector and minor vector species we have analyzed. Third, we confirmed that the centromeric region of chromosome *X* is critical to separate the M and S forms [21], [22], [23], [24], [29]. The centromere of chromosome *2L* was also reported to distinguish these two forms [18], [24], [26], [27], [28], [29]. Thus, taken together, our data and previous work point to the centromere regions as strong identifiers of different populations of the *A. gambiae* complex. Reduced recombination rates and a strong differentiation at the sequence level between the M and S forms have been reported near the *X* centromere region [33], [34]. It would be interesting to measure recombination rates near the *2L*- and *3^rd^* chromosome centromeres. Regions adjacent to centromeres are known to experience less recombination in several species, and it has recently been proposed that low recombination rates can facilitate the accumulation and maintenance of isolation genes in partially isolated populations.

Migrants, or admixed individuals, were found mostly between M and S molecular forms and between *A. gambiae s.s.* and *A. arabiensis*, reflecting the genetic proximity of these populations. The detected frequencies between M and S forms (0.6–3.9%) are similar to those (0.3–5.6%) estimated or found previously [17], [37], [38], [39]. The migrant frequencies between *A. gambiae s.s.* and *A. arabiensis* 0.5–4.7% are much higher than those observed in the field previously (0.01–0.2%) [8], [40]. The presence of admixed individuals between *A. gambiae s.s.* and *A. merus* (2.0%) further supports the previously reported close relationship between these two species [5], [9], [14].

In summary, about 30% of the detected admixed individuals between the M and S forms of *A. gambiae s.s., A. arabiensis, A. merus and A. melas* are allopatric mosquitoes with a high level of possible geographic isolation, thus the contribution of introgression to the variations in our sample set is estimated to be 70%. This estimation might be higher than in reality as we cannot exclude the possibility that some of the observed sympatric migrants resulted from ancestral polymorphisms.

Variation among loci is an expected consequence of recent speciation events, because, following divergence, some loci will develop monophyletic patterns more quickly than others, simply by chance (lineage sorting) [1]. We have observed in *A. melas*, five loci are monomorphic, though in most cases the microsatellite loci were polymorphic in all species. This might be an indication of lineage sorting. However, we have observed different phylogenetic relationships within the *Xag* inversion, as well as near the centromere region of the *X* chromosome, contrary to those on the autosomal regions. These data suggested that genetic divergence might have been a consequence of introgressive hybridization as has been proposed [3], [14], as well as experimentally demonstrated for species in the *A. gambiae* species [12].

Introgressive hybridization is a well-supported hypothesis for the *A. gambiae s.s.*/*A. arabiensis* species pair [12], [15], [18], which is consistent with the preponderance of sympatric admixtures among these taxa. The observed sympatric and allopatric migrants may have contributed to the expansion of *A. gambiae s.s.* across different ecological settings over Africa, in much the same way that hybrid sunflower species were found to occupy extreme environmental settings [41]. As a consequence, the *A. gambiae* species complex appears to be highly dynamic: continuous speciation occurs in situations where closely related taxa (M- and S-forms) are undergoing incipient speciation [26], while introgressive hybridization besides the lineage sorting of ancestral polymorphism among the taxa may provide traits that make these mosquitoes such a menace in Africa.

## Materials and Methods

### Mosquito samples

A total of 414 adult female mosquitoes were collected from several locations in Africa: Burkina Faso: Goundry; Mali: Selenkenyi (Sel), Soulouba (Soul) and Kokouna (Kn); Kenya: Kilifi and in Senegal (Table 1). Species were identified by PCR [42] and *A. gambiae s.s.* mosquitoes were further divided into M and S forms [43]. We grouped the 414 mosquitoes into nine populations ranging in sample size between 20 and 80, according to species identified by PCR and collection sites (Table 1).

### Cytogenetic localization

A bacterial artificial chromosome (BAC) library of *A. gambiae* (described in [44]) was screened by PCR with microsatellite-specific primers [36], [45]. Validated BAC clones were localized on squashed nurse cell polytene chromosomes of *A. gambiae* by *in situ* hybridization as described [46]. Hybridization images and illustrations of the detailed chromosomal location of each clone are available in the *Anopheles* database AnoDB (http://www.anobase.org/cgi-bin/insitu.pl).

### Genotyping

Mosquitoes were collected and genomic DNA was extracted as described [8]. Of the 42 microsatellite markers used in this study (see Table S1), 35 were used previously [21], [22], [36], [47], [48], [49], [50], and 7 were newly designed. Attempts to design microsatellite markers near the *2L* centromere failed because all flanking sequences were not unique when compared to the genome sequence. In this region, the microsatellite family does exist [51]. At least three tested markers have the same flanking regions as do markers near the *X* centromere region. Genotyping was carried out as described [36] with some modifications. PCRs were performed using all the markers. Each reaction contained 125 p*M* of each primer, 0.15 m*M* of each dNTP, 0.025 U/µl of Taq polymerase, and 0.3–0.9 ng/µl genomic DNA of a single mosquito. Cycling conditions were 94°C denaturation for 2 min, followed by 30–35 cycles of 94°C for 15 s, 56–68°C for 30s, and 72°C for 30 s, with a final 72°C extension of 10 min. Diluted (1/10) and multiplexed PCR products were detected using MegaBase capillary sequencer (Amersham Biosciences), and allele sizes for each individual at each locus were assigned and manually checked using the Genetic Profiler software (Amersham Biosciences).

### Statistical analysis

The genotyping data were evaluated using the software *STRUCTURE* [32]. *STRUCTURE* uses Bayesian clustering method to assign individuals probabilistically to populations based on multilocus genotype data. In this analysis, we did not use prior information about the origin of individuals. Various combinations of the number of populations *K* (1≤*K*≤10) and the simulation lengths (10^4^, 5×10^4^, 10^5^, 5×10^5^, 10^6^ and 2×10^6^ “Burn-ins” and “Repeats” each) were tested. The probability of each individual belonging to each of the *K* populations was plotted.

In addition, principal component analysis [52] and correspondence analysis [35], [53] were performed systematically on genotypes either for the 414 mosquitoes taken individually or for the corresponding nine populations genome-wide or on parts of the genome. The 262 *A. gambiae s.s.* mosquitoes and the corresponding five populations were further analysed by PCA and CA at all 42 microsatellite loci.

## Supporting Information

Figure S1 Allele frequency vs. size distributions of the X chromosome
(1.31 MB TIF)

Figure S2 Allele frequency vs. size distributions of the 2R chromosome arm
(1.09 MB TIF)

Figure S3 Allele frequency vs. size distributions of the 2R/2L chromosome arms
(1.12 MB TIF)

Figure S4 Allele frequency vs. size distributions of the 3R chromosome arm
(1.10 MB TIF)

Figure S5 Allele frequency vs. size distributions of the 3L chromosome arm
(1.11 MB TIF)

Figure S6 PCA results of 2D plots
(2.27 MB TIF)

Table S1 The 42 microsatellite markers used in this study
(0.21 MB DOC)

Table S2 Raw genotyping data of the 414 individuals at 42 markers. The 42 markers are listed in the first row, and the first two letters in their names (AG) are omitted. The 414 individuals of the *A. gambiae* complex were grouped into nine populations: molecular forms M (M1 and M2), and S (S1, S2 and S3) of *A. gambiae* s.s., *A. arabiensis* (A1 and A2), *A. merus* (R), and *A. melas* (L). Each individual of a population is assigned an ID, which consists of the population name followed by a serial number. The sizes of the two alleles at the same locus are given for each individual (numbers with the same ID and the same locus name), indicating either heterozygosity (two alleles have different sizes) or homozygosity (two alleles have the same size). “-9” stands for missing data.
(0.27 MB XLS)

Table S3 Variation of 42 microsatellite loci in nine populations of the *A. gambiae* complex
(0.11 MB DOC)

Table S4 Inferring the number of clusters for the Burkina Faso and Mali M molecular forms (104 individuals) at 42 loci
(0.03 MB DOC)
