# Supplementary figures and images for: Mosaic Genome Architecture of the Anopheles gambiae Species Complex
Source: PLoS One. 2007 Nov 28;2(11):e1249. doi: 10.1371/journal.pone.0001249 (PMC2082662; doi:10.1371/journal.pone.0001249)

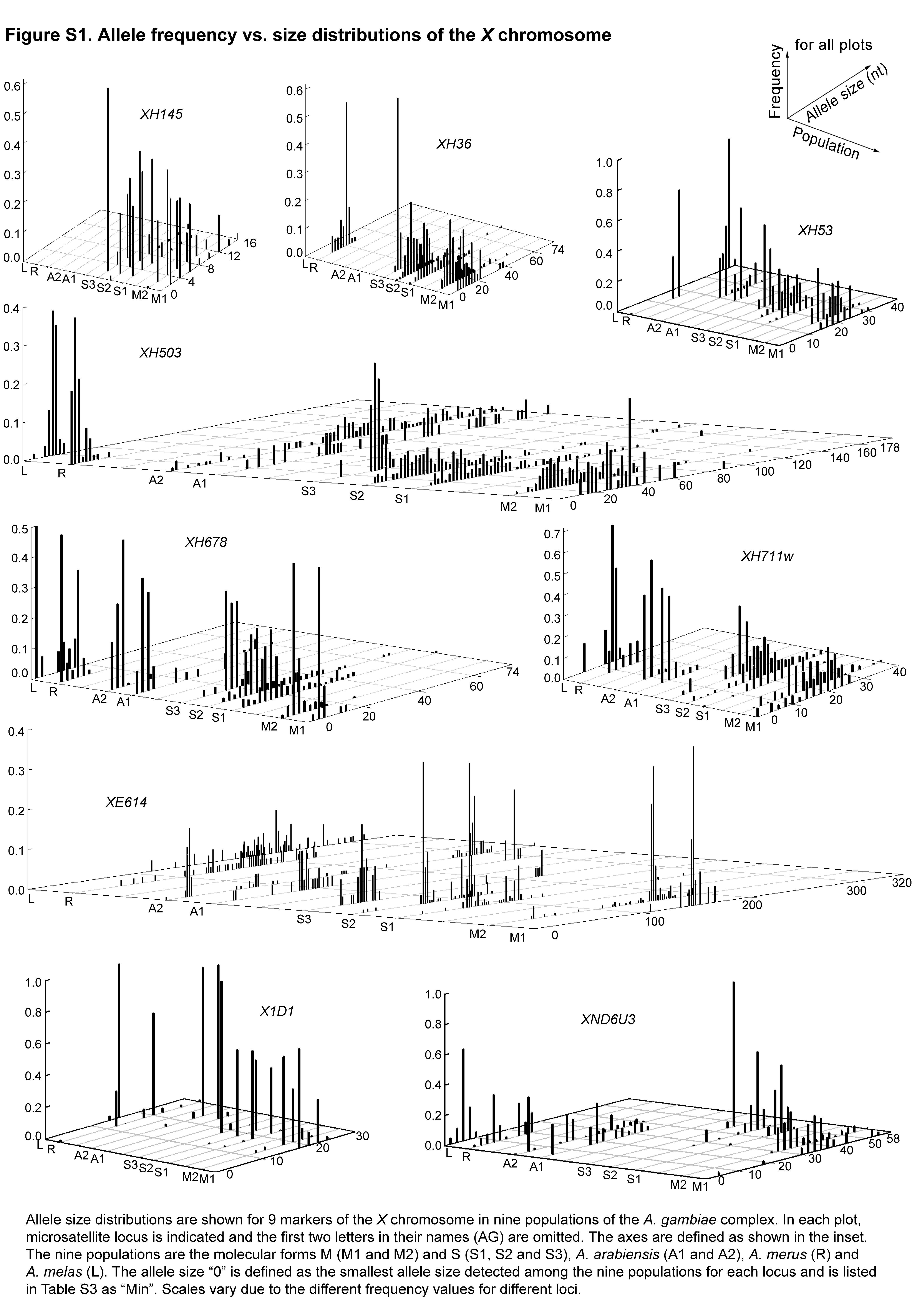

Supplement: Figure S1 — Allele frequency vs. size distributions of the X chromosome (1.31 MB TIF) [file pone.0001249.s001.tif]

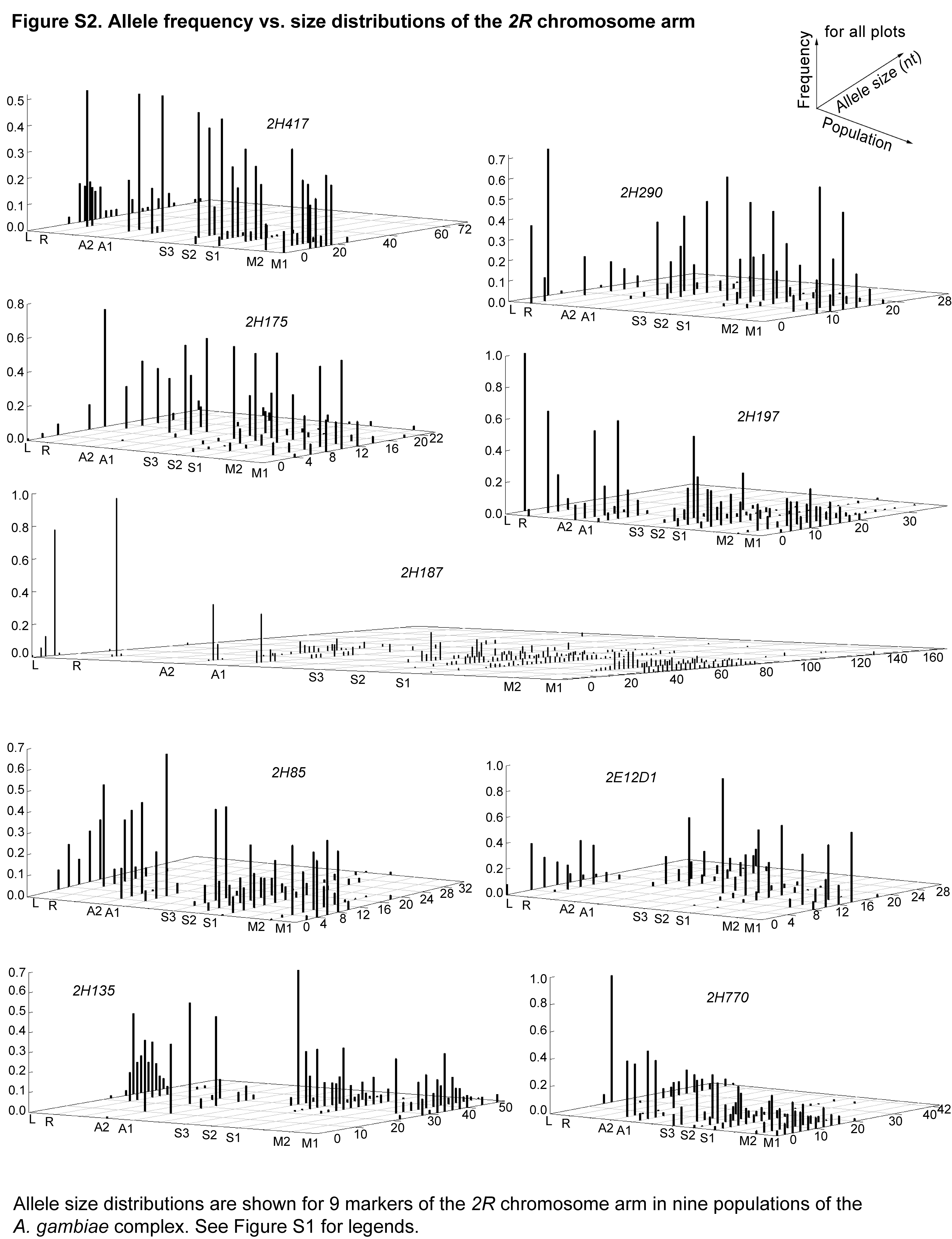

Supplement: Figure S2 — Allele frequency vs. size distributions of the 2R chromosome arm (1.09 MB TIF) [file pone.0001249.s002.tif]

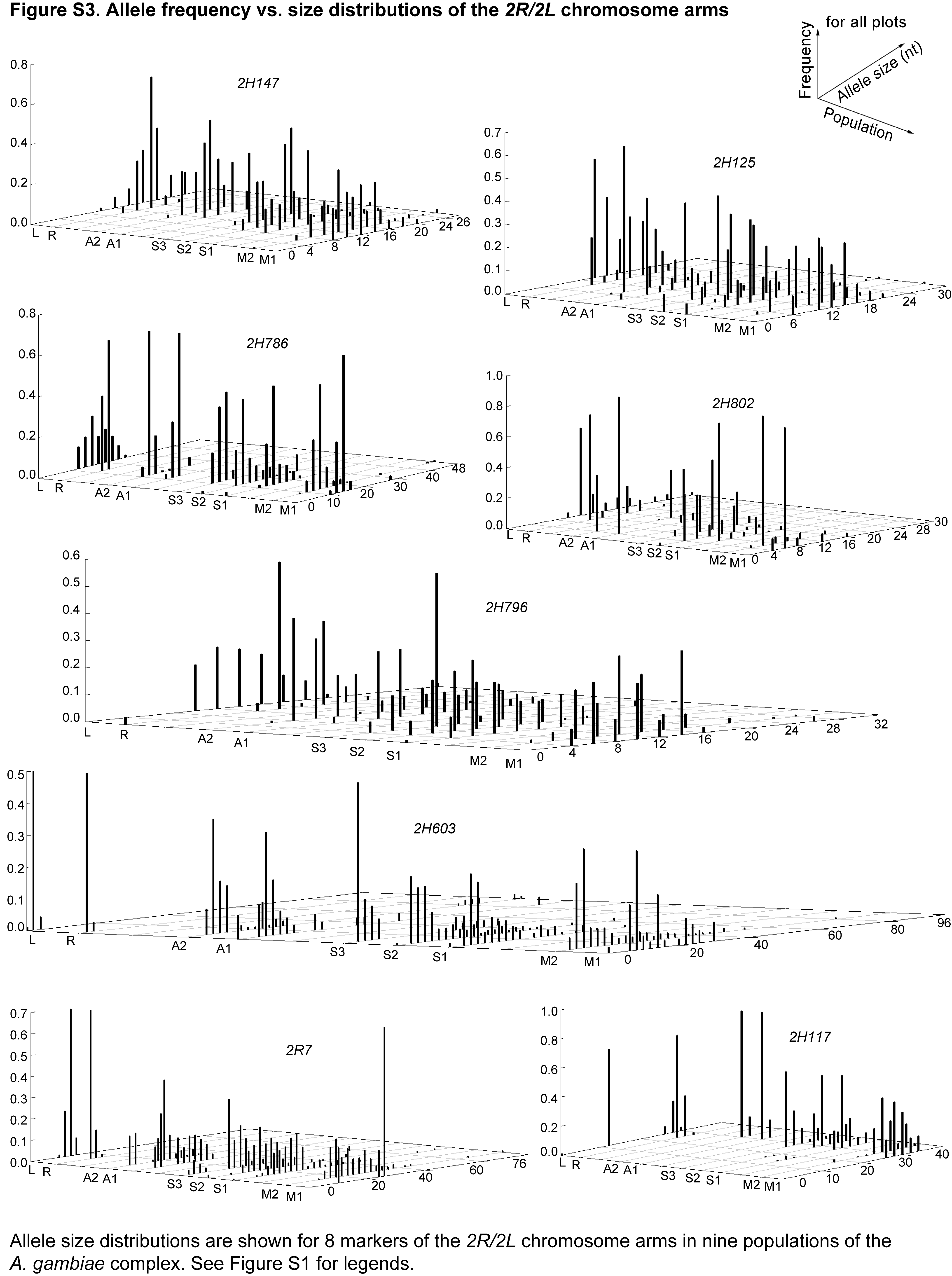

Supplement: Figure S3 — Allele frequency vs. size distributions of the 2R/2L chromosome arms (1.12 MB TIF) [file pone.0001249.s003.tif]

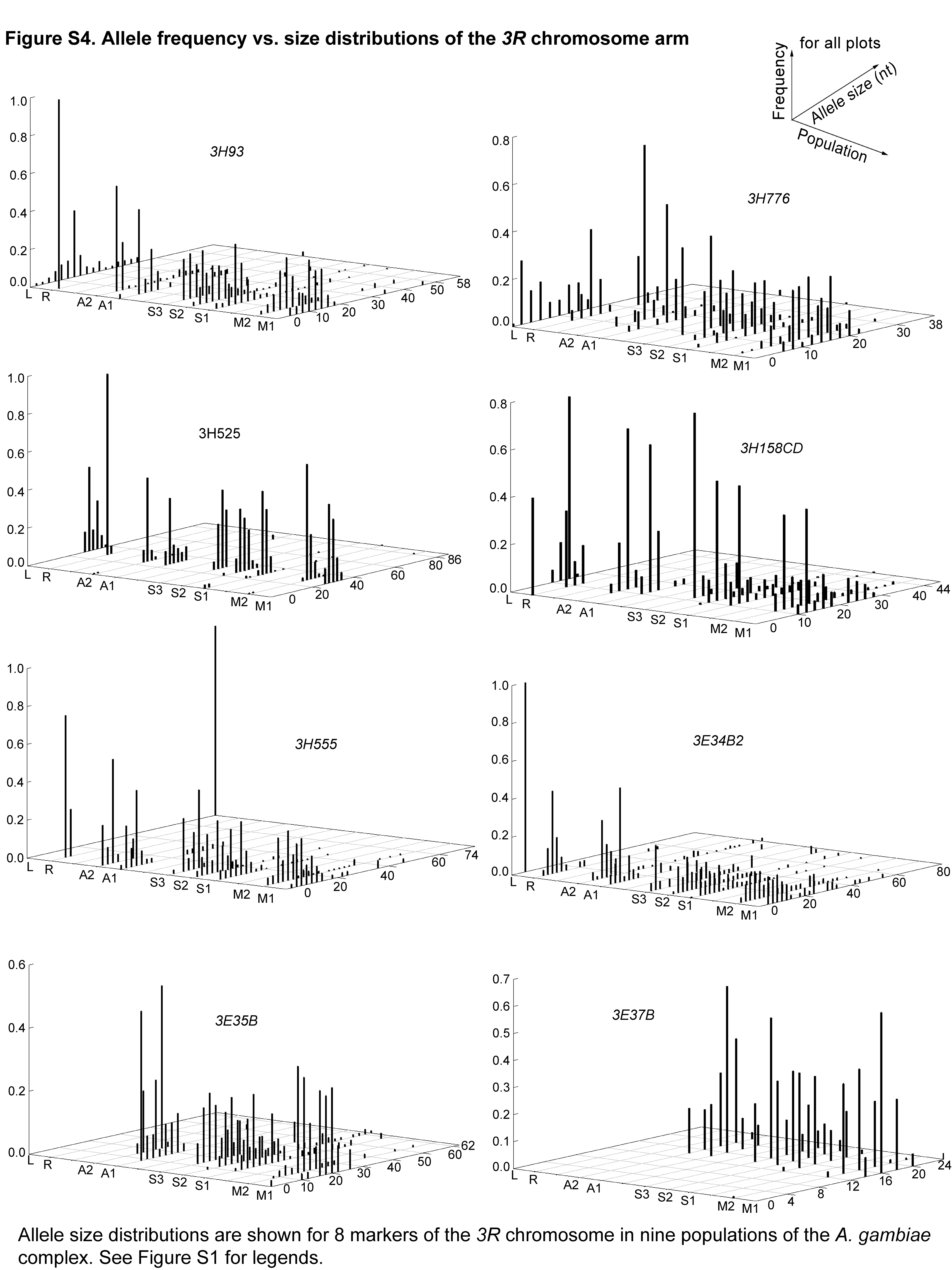

Supplement: Figure S4 — Allele frequency vs. size distributions of the 3R chromosome arm (1.10 MB TIF) [file pone.0001249.s004.tif]

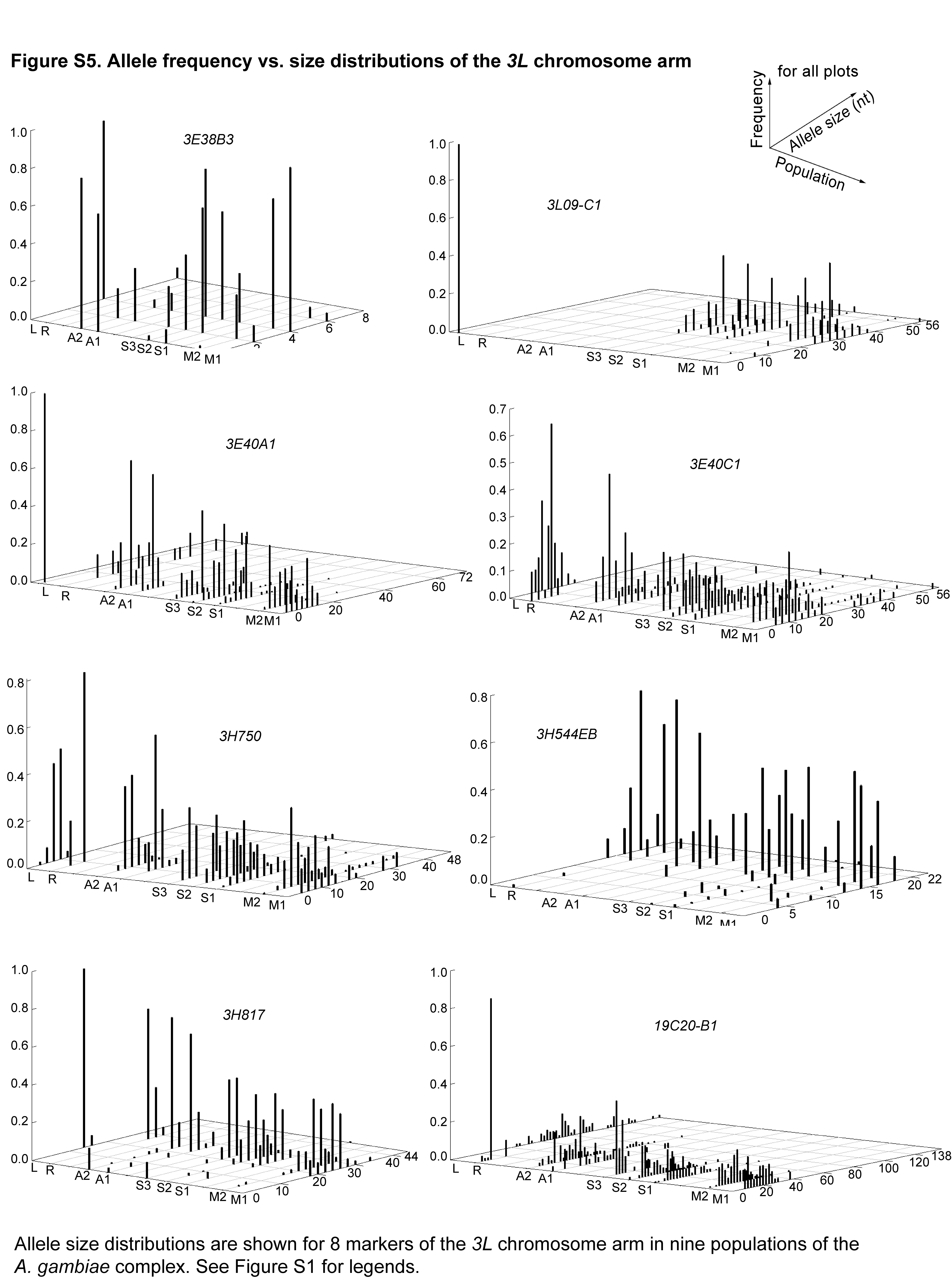

Supplement: Figure S5 — Allele frequency vs. size distributions of the 3L chromosome arm (1.11 MB TIF) [file pone.0001249.s005.tif]

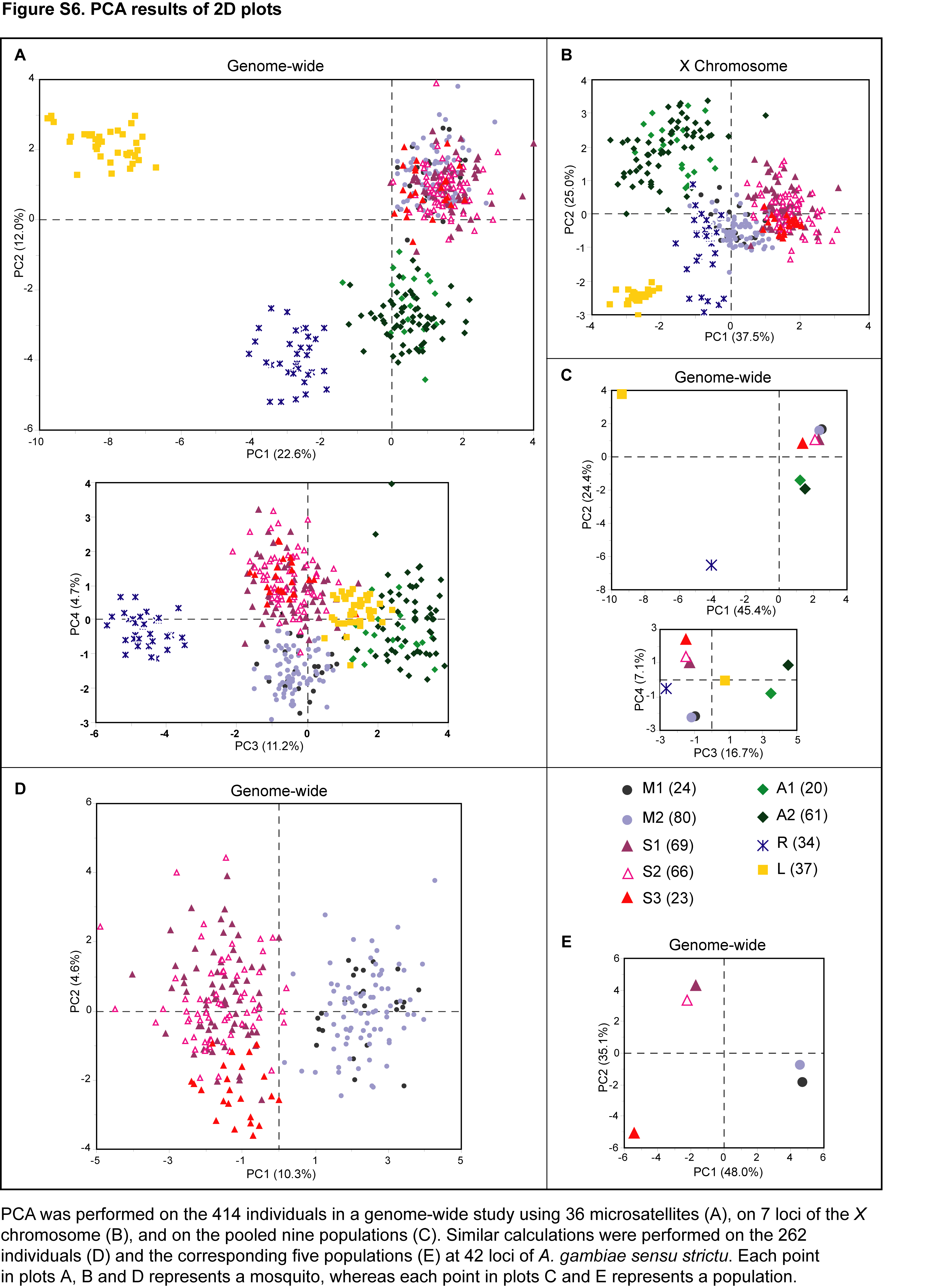

Supplement: Figure S6 — PCA results of 2D plots (2.27 MB TIF) [file pone.0001249.s006.tif]
