# Supplementary material for: Mosaic Genome Architecture of the Anopheles gambiae Species Complex
Source: PLoS One. 2007 Nov 28;2(11):e1249. doi: 10.1371/journal.pone.0001249 (PMC2082662; doi:10.1371/journal.pone.0001249)
Supplement: Table S1 — The 42 microsatellite markers used in this study (0.21 MB DOC) [file pone.0001249.s007.doc]

Table S1. The 42 microsatellite markers used in this study.

| Locus | Div | Sca | Genome locations | BAC clonefor *in situ* | Forward primer | Sequenced repeats | Reverse primer | References |
| --- | --- | --- | --- | --- | --- | --- | --- | --- |
| *X* |  |  |  |  |  |  |  |  |
| *AGXH145* | 4C | -- | -- | 1A18^I^ | TGGTGGAATGTGAGACACAG | (GT)11 | ATGATGGTCGATCCTTGTCC | [47] |
| *AGXH503* | 4B | 8846 | 1825845-928 | 10A05^II^ | AGGTTAGAGTGAGCAACCC | (GT)30 | GCACTGCATCTCTCCAATAC | [47] |
| *AGXH36* | 4B | 8846 | 1915346-533 | † | CGTATGTTTGCTAGGGGTGG | (GT)14 | GTCAAGAAATGGGCCACAGG | [47] |
| *AGXH53* | 3D | 8846 | 3630267-360 | † | GTTTCGGGGCTTGAGAAGTG | (GT)7 | CTTCACGTGGCTTTGCTGTG | [47] |
| AGXH711w | 2A | 8846 | 9884944-5074 | 20K19 | CCCACAGCAAAACGAGAAG | (GA)10+4 | GACAACTTGCATTTCACTATG | [47] |
| AGX1D1 | 1C | 8811 | 11656151-322 | - | GTTATCCACTGCGCATCATG | (GGT)_6_ | TAATGGTCCCAAATCGTTGC | [48] |
| AGXH678 | 5D | 8811 | 19168414-566 | 2N07 | CCTCTCCCCAGAATCGGTAC | (AG)7 | AAGAGCAGAAACAACCGCAG | [47] |
| *AGXND6U3* | 6 | 8811 | 20008488-706 | -- | CCCGGTTTTCGTGGTATCT | (CT)_20_GT(CT)_4_GT  (CT)_5_GT(CT)_7_ | ATTTCATGGGAAGGGAAGGA | [22] |
| *AGXE614* | 6 | 7654 | 9430903-31192 | 10D02 | CCAGCAGTCGTCGGGGCAAG | (CAG)7+13+4+17 | CGCTGCAGTCGTGGAGGAAG | [21] |
| *2R* |  |  |  |  |  |  |  |  |
| *AG2H417* | 7B | 8987 | 2419902-982 | 11A22 | GTGCAGGAGATGTTCCTACC | (GT)9 | GATGTGGTGTTTAGGCCCAG | [47] |
| *AG2H290* | 8C | 8987 | 6330491-579 | 17C08 | GGCGTGTGCTGTGCTCCC | (GT)9 | ACGCAATTTTTGCCTCAGCG | [47] |
| *AG2H175* | 8D | 8987 | 6554063-159 | -- | AGGAGCTGCATAATTCACGC | (CA)8 | AGAAGCATTGCCCGCATTCC | [47] |
| *AG2H197* | 9A | 8987 | 10405738-826 | 16N02 | TACCTCTGTGTTCGGTTTCC | (GT)8 | GGTGGTATGGCGATGGAAGG | [47] |
| *AG2H187* | 11A | 8799 | 17806585-669 | 25L17 | CCGGAGCAGAGATAAACAGC | (GT)48 | CACAGCGTACACCTAATGC | [47] |
| *AG2H85* | 11A | 8799 | 17873355-500 | 6K01 | ATTTATCATACGGCGCCCAC | (GT)11 | TTGAAAGGTTGCAACGAGGCG | [47] |
| *AG2E12D1* | 12D | 8859 | 24861437-565 | -- | TCACACAGTGTATGCGCAAG | (GT)3+7 | TTGATGGTCGGCTCAGTAAA | New |
| *AG2H135* | 14D | 8879 | 34371317-96 | 5G23 | TCATGCACTGTTTGCTCGGC | (GT)7 | CTGCCCCATTCAATTGCAGC | [47] |
| *AG2H770* | 15D | 8888 | 40443556-721 | -- | CAAGATGGAGGCGCATGATC | (GT)8+4 | GCGTTCCATCGAAATCAGAC | [47] |
| AG2H147 | 16A | 8888 | 41979597-768 | -- | CTGCTGTTGCTGCCAAAATG | (GT)8 | AGCTTCACGGAAAGCAAAGG | [47] |
| *AG2H125* | 17A | 8805 | 45660190-281 | -- | AGGAGCATAACACATCGCCC | (GT)11 | CGCTCGTCAAAGAAACTGGC | [47] |
| *AG2H786* | 17C | 8817 | 48304947-5040 | 3I17 / 8F02 | TGTGAAGCATTTCCTTGGCG | (GT)11 | TGCCCTTGAGTCGAGGTAGC | [47] |
| 2L |  |  |  |  |  |  |  |  |
| AG2H796 | 22C | 8960 | 16729412-98 | -- | CTTTGCCATTGCACGGTCCC | (GT)10 | TTCGGCTCCGCTCACTCAAC | [47] |
| AG2H802 | 24C | 8960 | 30876135-263 | -- | TTTGGTGAGGGGTTTGTTCC | (TG)31+24 | GGCAATTCATTCGTCACACC | [47] |
| AG2R7 | 27A | 8807 | 38651672-772 | † | ATGGTTTCTGTGGAACGACC | (CA)9 | CTAAAGGTCCTGGTTGTTCG | [49] |
| *AG2H603* | 26C | 8807 | 42014745-850 | 20L10 | TGCACCGTTGATGCACATGC | (GT)8 | GTGGACGATGTGAAAGATAAGG | [47] |
| *AG2H117* | 28D | 8807 | 48951720-824 | 22L01 | CGGAACGCACGGAACAATTG | (GT)6+4 | CGTTGCAGATTTCCCAAACG | [47] |

**Table S1, continue**d

| Locus | Div | Sca | Genome locations | BAC clonefor *in situ* | Forward primer | Sequenced repeats | Reverse primer | References |
| --- | --- | --- | --- | --- | --- | --- | --- | --- |
| *3R* |  |  |  |  |  |  |  |  |
| AG3H93 | 29A | 8964 | 1022141-300 | † | TCCCCAGCTCACCCTTCAAG | (GT)4+7 | GGTTGCATGTTTGGATAGCG | [47] |
| *AG3H776* | 29C | 8964 | 2439524-617 | 32B02 | TGCGGATCATAATCGAGTCC | (GT)7 | TCACAAACACGCAACGAGTC | [47] |
| *AG3H525* | 30D | 8964 | 11072776-861 | 30E13 | AAGGTGACGCTTACGTACGG | (GT)7 | ATCATCAAAAACCTGCCGCC | [47] |
| *AG3H158CD* | 32D | 8984 | 22997125-213 | 23K23/19K19 | CACGATCAATCAATCGATCG | (GT)12 | CAAGACGATGGTGTACACGT | [21] |
| *AG3H555* | 33B | 8984 | 21302466-554 | 7C24^III^ | GCAGAGACACTTTCCGAAAC | (GT)8 | TGTCAACCCACATTTTGCGC | [47] |
| *AG3E34B2* | 34B | 8839 | 34193977-4151 |  | TCGGTTGGGTACAAGAAAGG | (GT)14 | GGTTTGCCATTCTGCTTTGT | New |
| *AG3E35B* | 35B | 8980 | 39494052-216 | -- | TCGAGTGCAGTGGAGAGTTG | (GT)15 | CAAACCGGAATGGTCTCTGT | New |
| *AG3E37B* | 37B | 8980 | 50439481-608 | -- | TGGAATTTGGACTCGTGTGA | (CA)9 | AACGTGTCTCGCTACTGCAC | New |
| *3L* |  |  |  |  |  |  |  |  |
| *AG3E38B3* | 38B | 8849 | 3281685-899 | -- | ATCCGGAATAGCGCACATAC | (GT)7 | TGGCACGAGATATCAAACGA | New |
| *3L09-C1* | 38B | 8933 | 5123942-4110 | -- | GAAACTTCCCTGAATTTCCGC | (CA)10+8 | ATGGAGGCGACTAAACGTTGC | [50] |
| *AG3E40A1* | 39C | 8979 | 10802338-543 | -- | CGCATCAAAAGTGAACATGC | (GT)10 | GAATTATTCCCGCCAGTGTG | New |
| *AG3E40C1* | 40A | 8823 | 12526816-999 | -- | TATTCAAACCGGACGTAGGC | (GT)19 | AGCAAACGAGCGTTGAACTT | New |
| *AG3H750* | 41A | 8816 | 17377655-755 | 29A12 | GCAAAAAAGCTTCTCCCC | (GT)8 | TTAGCTACCGTCGACGCTTC | [47] |
| *AG3H544EB* | 44B | 8816 | 21119886-20045 | 3E18 | GCTCGTCGATCCTGATCGAA | (GT)7 | CTCGCCCTTTTCCCCTTACC | [21] |
| *AG3H817* | 44B | 8986 | 31854973-5102 | † | ACTGGTCCGTTGCTGCGCG | (GT)8 | ATGAGTGAATGGTGCGCTGG | [47] |
| *19C20-B1* | 46C | 8986 | 41558503-660 | -- | ACCAAGTGCTCTTTGGGTTG | (TG)15 | ACGAATGCTGGACAATGTGA | [50] |

The list of markers is shown in the first column. The cytogenetic locations (Div) are shown in column 2. The Sca. represents last four digits of scaffold number, preceded in all cases by AAAB0100 is listed in column 3. Genome locations are shown in column 4. BAC clones containing each microsatellite marker and used for *in situ* hybridization are listed in column 5. The type and number of nucleotide repeats in the original sequenced allele are shown in column 7, and the forward and reverse primers are shown in column 6 and 8, respectively. The last column shows the references, in which the markers were first introduced

I The BAC hybridizes to a second site at 30B.

II A strong single signal at 4B (compatible with the genetic map) and 15 weaker signals on chromosome *3* (e.g. 29C, 31C, 42A, 44D etc.) were observed using the entire BAC clone; a single band at 4B resulted from using a 0.5 kb DNA fragment bearing the microsatellite.

III The BAC hybridizes to a second site at 37D.

† Mapped using PCR fragments or M13 clones bearing the microsatellite [47] [49].
