# Supplementary material for: Mosaic Genome Architecture of the Anopheles gambiae Species Complex
Source: PLoS One. 2007 Nov 28;2(11):e1249. doi: 10.1371/journal.pone.0001249 (PMC2082662; doi:10.1371/journal.pone.0001249)
Supplement: Table S3 — Variation of 42 microsatellite loci in nine populations of the A. gambiae complex (0.11 MB DOC) [file pone.0001249.s009.doc]

**Table S3. Variation of 42 microsatellite loci in nine populations of the *A. gambiae* complex**

| Locus | Min | Relative allele size range  M1 M2 S1 S2 S3 A1 A2 R L  (24) (80) (69) (66) (23) (20) (61) (34) (37) | Number of alleles  M1 M2 S1 S2 S3 A1 A2 R L  (24) (80) (69) (66) (23) (20) (61) (34) (37) | Heterozygosity (%)  M1 M2 S1 S2 S3 A1 A2 R L  (24) (80) (69) (66) (23) (20) (61) (34) (37) |
| --- | --- | --- | --- | --- |
| *X* |  |  |  |  |
| *AGXH145* | 65 | 2-16 0-12 2-14 0-16 2-6 -- -- -- -- | 8 7 7 9 3 -- -- -- -- | 71 69 70 73 35 -- -- -- -- |
| *AGXH503* | 66 | 12-88 4-128 10-154 6-168 14-66 16-66 2-178 0-20 6-22 | 15 34 42 41 10 28 52 9 7 | 42 80 80 91 35 75 60 68 39 |
| *AGXH36* | 182 | 6-38 4-50 0-42 0-74 8-24 -- -- 10-26 -- | 11 17 19 21 4 -- -- 9 -- | 83 81 91 89 26 -- -- 71 -- |
| *AGXH53* | 72 | 14-30 16-32 12-30 14-30 18-26 20-32 20-34 0-16 36-40 | 8 9 10 9 4 5 8 3 3 | 75 71 83 68 57 55 51 74 30 |
| *AGXH711w* | 110 | 0-26 4-46 0-42 2-56 4-26 8-18 8-14 8-18 6-16 | 13 19 15 23 8 6 4 6 4 | 71 83 80 91 70 50 48 75 49 |
| *AGX1D1* | 156 | 18-24 12-24 12-24 12-22 18-20 24-26 16-26 0-30 14-16 | 4 6 5 3 2 2 3 7 2 | 38 48 54 48 9 10 16 38 0 |
| *AGXH678* | 148 | 2-14 0-26 4-74 6-66 8-24 2-34 2-60 2-12 2-16 | 5 10 22 23 7 8 9 6 6 | 29 34 86 92 65 60 39 91 65 |
| *AGXND6U3* | 176 | 4-54 28-58 30-50 26-50 44-46 4-34 0-40 2-20 2-10 | 11 15 7 9 2 10 15 9 5 | 81 96 68 69 9 71 79 40 51 |
| *AGXE614* | 172 | 108-168 40-190 40-268 12-208 34-222 20-288 0-304 44-230 116-324 | 9 29 25 24 17 20 34 38 36 | 50 63 68 91 74 83 100 65 68 |
| *2R* |  |  |  |  |
| *AG2H417* | 69 | 0-24 2-20 2-30 2-20 12-18 12-16 12- 16 14-16 16-72 | 6 6 9 6 4 3 2 2 18 | 54 71 71 77 52 30 26 26 89 |
| *AG2H290* | 82 | 8-18 8-18 8-22 8-22 12-16 10-20 8-28 0-16 6-14 | 6 6 7 8 3 6 9 6 3 | 71 69 67 73 48 75 69 62 3 |
| *AG2H175* | 120 | 4-18 4-22 4-22 2-22 6-22 8-20 4-22 10-16 0-10 | 7 7 8 10 6 5 5 4 6 | 42 94 48 35 26 30 61 76 11 |
| *AG2H197* | 79 | 4-20 0-36 2-38 2-26 4-22 0-10 0-10 0-8 4 | 9 18 17 12 7 6 6 4 1 | 67 84 67 74 61 40 51 35 0 |
| *AG2H187* | 58 | 36-136 34-136 6-144 20-148 38-108 14-156 14-90 14-18 0-68 | 26 33 43 39 19 18 21 3 7 | 88 81 86 91 70 56 67 6 32 |
| *AG2H85* | 143 | 2-18 0-18 0-32 2-32 4-12 4-10 4-10 10-22 6-14 | 6 10 14 15 5 4 4 7 5 | 67 73 75 78 43 30 36 53 97 |
| *AG2E12D1* | 120 | 8-18 8-16 4-20 4-20 12-14 14-26 12-28 4-14 0-14 | 5 5 8 8 2 7 9 6 7 | 78 92 55 54 22 57 72 12 72 |
| *AG2H135* | 71 | 22-48 28-50 22-48 22-44 28-34 10-32 0-30 20-40 22-38 | 10 12 13 12 4 6 5 8 8 | 46 70 70 80 48 60 64 88 59 |
| *AG2H770* | 156 | 0-20 0-18 0-36 0-36 0-16 2-8 4-16 18-42 14-16 | 10 10 13 15 6 4 5 13 2 | 96 95 90 83 74 47 69 68 8 |
| *AG2H147* | 164 | 8-20 0-26 8-24 8-26 10-18 12-18 10-18 10-18 10-22 | 7 13 10 10 5 5 6 6 8 | 32 87 45 57 70 50 76 44 69 |
| *AG2H125* | 82 | 6-20 4-30 0-30 0-18 8-16 4-18 6-18 10-22 14-18 | 7 9 13 8 5 7 6 7 3 | 63 91 66 80 73 74 35 65 86 |
| *AG2H786* | 81 | 10-28 8-48 0-28 0-28 10-16 10-14 10-24 12-14 12-26 | 5 11 10 19 4 3 6 2 8 | 50 50 58 65 61 45 21 50 49 |
| *2L* |  |  |  |  |
| *AG2H796* | 76 | 4-26 4-28 0-16 4-20 4-28 8-26 6-24 12-22 10-32 | 8 11 8 9 7 9 9 7 8 | 58 86 67 83 74 90 78 56 91 |
| *AG2H802* | 126 | 4-16 4-16 6-16 0-16 6-18 4-16 4-18 10-22 10-30 | 5 6 6 8 5 3 4 5 9 | 26 85 25 38 43 20 69 50 50 |
| *AG2R7* | 110 | 10-26 12-76 0-70 0-48 2-58 8-28 8-32 12-16 10-16 | 3 18 20 15 11 9 11 3 4 | 0 80 83 70 70 70 66 24 8 |
| *AG2H603* | 103 | 0-30 4-80 0-50 0-62 4-44 0-86 6-96 2-4 0-4 | 12 23 19 22 9 13 17 2 3 | 67 66 74 86 57 90 89 6 11 |
| *AG2H117* | 72 | 26-34 18-38 14-38 16-38 26-36 30-32 30-32 24-28 0-30 | 5 9 9 8 6 2 2 3 5 | 79 75 61 61 61 15 23 21 54 |

**Table S3, continue**d

| Locus | Min | Relative allele size range  M1 M2 S1 S2 S3 A1 A2 R L  (24) (80) (69) (66) (23) (20) (61) (34) (37) | Number of alleles  M1 M2 S1 S2 S3 A1 A2 R L  (24) (80) (69) (66) (23) (20) (61) (34)(37) | Heterozygosity (%)  M1 M2 S1 S2 S3 A1 A2 R L  (24) (80) (69) (66) (23) (20) (61) (34) (37) |
| --- | --- | --- | --- | --- |
| *3R* |  |  |  |  |
| *AG3H93* | 142 | 4-46 2-44 2-56 2-56 6-46 0-58 6-36 2 2-34 | 10 13 18 18 8 10 14 1 17 | 92 65 61 85 48 75 43 0 84 |
| *AG3H776* | 82 | 6-22 2-30 2-34 6-38 8-18 6-38 8-30 8-26 0-16 | 9 13 13 13 4 11 10 8 9 | 63 79 75 80 48 70 33 76 81 |
| *AG3H525* | 57 | 26-34 0-80 0-86 20-66 26-32 24-76 0-38 28-30 28-38 | 5 9 13 9 4 7 7 2 6 | 67 51 57 62 61 30 23 9 65 |
| *AG3H158CD* | 77 | 10-28 10-38 10-44 8-36 12-22 10-20 8-14 0-12 10-16 | 9 14 14 13 4 5 4 4 4 | 29 74 58 73 35 50 34 59 30 |
| *AG3H555* | 79 | 2-46 2-54 2-44 2-50 2-14 0-40 2-14 6-8 74 | 12 20 16 14 6 12 5 2 1 | 92 79 68 88 78 90 66 44 0 |
| *AG3E34B2* | 157 | 4-54 2-62 0-74 2-74 2-74 4-70 6-80 4-54 6 | 17 23 22 25 16 10 22 10 1 | 79 94 70 72 91 58 74 50 0 |
| *AG3E35B* | 141 | 0-26 8-54 10-58 8-62 12-44 8-22 14-50 -- -- | 8 14 18 23 9 8 6 -- -- | 54 87 56 64 65 55 26 -- -- |
| *AG3E37B* | 108 | 14-24 0-22 12-22 14-22 14-22 14-20 14-22 -- -- | 5 7 6 5 5 4 5 -- -- | 33 88 46 54 48 46 61 -- -- |
| *3L* |  |  |  |  |
| *AG3E38B3* | 215 | 2-6 0-6 0-4 0-4 2-4 0-4 0-4 -- 4-8 | 3 4 3 3 2 3 3 -- 2 | 26 79 15 7 35 19 9 -- 11 |
| *3L09-C1* | 137 | 8-52 8-52 22-54 22-56 20-38 -- -- -- 0 | 10 13 14 13 9 -- -- -- 1 | 87 95 80 65 73 -- -- -- 0 |
| *AG3E40A1* | 192 | 2-14 2-30 2-44 0-34 2-28 6-14 4-12 14-46 2 | 7 9 14 13 9 5 4 11 1 | 91 92 78 73 74 53 52 38 0 |
| *AG3E40C1* | 153 | 6-54 6-50 2-56 2-56 6-54 6-48 6-30 0-8 8-20 | 15 20 22 20 9 15 11 5 7 | 87 95 87 83 83 74 71 53 83 |
| *AG3H750* | 78 | 4-32 0-38 2-38 4-32 4-48 8-16 6-18 6-10 4-12 | 10 17 13 10 7 5 7 2 5 | 63 74 59 65 52 60 57 21 38 |
| *AG3H544EB* | 127 | 20-34 22-34 16-38 16-38 30-34 26-34 30-34 16-36 30-36 | 6 6 12 9 3 4 3 6 4 | 50 31 25 48 26 35 38 59 38 |
| *AG3H817* | 105 | 18-34 4-30 6-44 6-30 0-24 0-28 0-24 26-30 14-16 | 8 10 8 9 4 6 6 3 2 | 71 66 71 71 65 45 46 53 5 |
| *19C20-B1* | 139 | 8-38 2-54 0-68 0-58 8-30 0-74 8-102 4-20 44-138 | 13 19 23 23 7 17 29 5 29 | 96 95 87 89 65 63 67 29 64 |
| Average |  |  | 9 14 15 14 7 8 10 7 7 | 62 77 66 71 54 54 53 47 42 |

42 markers were genotyped for 414 individuals of the *A. gambiae* complex (with the number of mosquitoes analyzed shown in parentheses): molecular forms M (M1 and M2), and S (S1, S2 and S3) of *A. gambiae*, *A. arabiensis* (A1 and A2), *A. merus* (R), and *A. melas* (L). In column 2, “Min” is defined as the smallest allele size detected among the 414 individuals for each locus. The relative allele size range, number of alleles and observed heterozygosity are shown in columns 3 and 5. -- = no PCR products detected in this species. The average number of alleles and heterozygosity for each group at all 42 loci are listed in the bottom row.
