# Supplementary material for: Mosaic Genome Architecture of the Anopheles gambiae Species Complex
Source: PLoS One. 2007 Nov 28;2(11):e1249. doi: 10.1371/journal.pone.0001249 (PMC2082662; doi:10.1371/journal.pone.0001249)
Supplement: Table S4 — Inferring the number of clusters for the Burkina Faso and Mali M molecular forms (104 individuals) at 42 loci (0.03 MB DOC) [file pone.0001249.s010.doc]

**Table S4. Inferring the number of clusters for the Burkina Faso and Mali M molecular forms (104 individuals) at 42 loci.**

| *K* | *ln(P(X/K))* | *P(K/X)* |
| --- | --- | --- |
| 1 | -16762 | ~ 0 |
| **2** | **-16664** | **1** |
| 3 | -16929 | ~ 0 |
| 4 | -17405 | ~ 0 |
| 5 | -17562 | ~ 0 |

For each *K* number of clusters of the *X* genotypes, the estimated values of *ln(P(X/K))* are listed for 5×10^5^ “Burn-ins” and “Repeats”, respectively.
